# The long-term impact of the earthquake on substance use

**DOI:** 10.1186/s12245-022-00449-x

**Published:** 2022-09-05

**Authors:** Hadis Amiri, Sevda Riyahifar, Nouzar Nakhaee, Mahmoud Nekoei-Moghadam

**Affiliations:** 1 Health in Disasters and Emergencies Research Center, Institute for Futures Studies in Health, Kerman University of Medical Sciences, Kerman, Iran; 2 Department of Biostatistics, School of Public Health, University of Medical Sciences, Tehran, Iran; 3 Health Services Management Research Center, Institute for Futures Studies in Health, Kerman University of Medical Sciences, Kerman, Iran; 4 Department of Health in Emergency and Disasters, School of Healthcare Management and Medical Information, Kerman University of Medical Sciences, Kerman, Iran

**Keywords:** Earthquake, Substance abuse, Drug use, Tobacco, Sedatives

## Abstract

Earthquake is associated with several health conditions such as posttraumatic stress disorder (PTSD), depression, and cardiovascular disease. However, the association between earthquakes and substance use has been less studied to date. We conducted a historical cohort study 17 years after the Bam earthquake by enrolling 818 households using multi-stage cluster sampling. The sample consisted of earthquake-exposed and non-exposed citizens. The ASSIST screening test was used to determine substance use. Logistic regression analysis was used to evaluate the association of variables of interest with substance use. Nearly 60% of the study subjects were female and the mean ± SD age of the sample was 46.6 ± 11.5 years. The prevalence of tobacco, alcohol, and other drug use in the exposed group was 19.5%, 24.9%, and 21.6%, respectively. The corresponding figures in the non-exposed group were 15.6%, 19.3%, and 20.1%, respectively (*P* > 0.05). The logistic regression model found no association between the history of earthquake exposure and the risk of any current drug use. Our results showed those 17 years after the Bam earthquake, there was no relationship between earthquake exposure and substance use.

## Introduction

According to the Centre for Research on the Epidemiology of Disasters, about 200 million people are annually exposed to disasters worldwide [1]. Natural disasters frequently impact Iranian cities like many other countries, leading to a high degree of casualty and billions of dollars of damage to properties and infrastructure [2]. Earthquakes are one of the most destructive natural disasters. On the other hand, narcotic and alcohol consumption is dramatically rising worldwide and is threatening different countries with a negative impact on health, increasing crimes, preventing productivity, destroying relationships, diminishing social and moral values, and hindering the overall progress of communities [3].

Attention to natural disasters and their behavioral outcomes motivated a growing body of literature on the effects of exposure to disasters and substance use [4, 5]. The Substance Abuse and Mental Health Services Administration (SAMHSA) has confirmed the correlation between exposure to disasters and the risk of substance use [6]. Previous studies also affirm a strong relationship between substance use by adolescents and being exposed to natural or human-made disasters [7, 8]. According to a study using self-report measures conducted 13 months before and 7 and 19 months after Hurricane Rita on the coasts of the USA, people who had no history of substance or alcohol consumption before the hurricane started smoking cigarettes (15%), drinking (25%), and smoking marijuana (8–9%) afterwards [9]. Based on another study, those who were directly exposed to Hurricane Ike in Galveston Island (i.e., those who had not evacuated the region before the hurricane) reported higher consumption of alcohol, hash, and cocaine compared to those who were not directly exposed to it (i.e., those who had evacuated before the hurricane) [10].

Disasters cause different damages at the personal and social levels, thereby causing a level of stress that may provide uncontrollable [11]. People show different coping reactions in response to stress, including the continued use of substances that potentially contributes to substance abuse disorder [6]. Note that exposure to disasters in the short term is generally associated with a rise in substance use, but this effect may be transient, but little is known about such an effect over a long period (more than 15 years).

Kerman Province runs a high risk of earthquakes among Iranian provinces [12] and has experienced several earthquakes due to its geographical location, e.g., 2005 Zarand [13], 2011 Kahnouj [14], and 1981 Golbaf [15]. The deadliest natural event occurred in 2003, when an earthquake at the depth of 8 km hit the city of Bam, Kerman Province, and killed 41,000 people [16]. Another cause of concern in Kerman Province is its location on the narcotic smuggling path. Kerman is located near Afghanistan, the largest narcotic producer and exporter in the world. As such, access to narcotics is easier for Kermani citizens.

To the best of our knowledge, no study has assessed the long-term effect of exposure to the earthquake on substance use. Thus, this study aimed to examine the effect of the Bam earthquake on substance use 17 years after the event.

## Method

The researchers collected the data in a historic cohort study 17 years after the Bam earthquake using multi-stage cluster sampling. With a population of about 130,000, Bam is located in the southeast of Kerman Province. On 26 December 2003, a magnitude 6.2 earthquake struck the city and killed 41,000 people. The sample comprised two groups of exposed and non-exposed. The exposed group consisted of people who were present in Bam now of the earthquake and objectively experienced it. The inclusion criteria for the non-exposed group were having resided in Bam for a maximum of 2 years after the earthquake (to ensure relative homogeneity in terms of the sociocultural context of living in Bam) and residing there until the time of the study. People who were at least 15 years of age at the time of the earthquake were included. By asking “Which of the following substances have you ever consumed?” the consumption of drugs was categorized to three subgroups tobacco, alcohol, and other drugs (opioids, marijuana, methamphetamines, and sedatives). Demographic variables including sex and age, level of education, occupation, place of residence at the time of the earthquake, age at marriage, and marital status were also collected. Regarding the ameliorating role of the post-traumatic growth (PTG) on the consequences of earthquakes [17], we measured the level of PTG too. To neutralize recall bias, those with mental conditions who could not concentrate or respond were excluded. There was no follow-up bias since this was a historic cohort study [18]. The anonymous questionnaire took about 20 min to complete. Participants’ written consent to participate in the study was sought at the start of the questionnaire.

According to the post hoc analysis calculated by G-Power software (Version 3.1.9.2, Düsseldorf University, Düsseldorf, Germany), we achieved a power of 0.82 with 818 subjects, an alpha level of 0.05, and an effect size of 0.1 [19] using drug use as the primary outcome measure. The multistage cluster sampling was based on the socioeconomic status of their living region. At first, based on the socioeconomic status of different regions of the city, 12 clusters were selected. Then, by using the probability proportional to size (PPS) sampling, the clusters were selected. More clusters were chosen from categories with more households and neighborhoods. The number of households per cluster is recommended to be 20–40. Herein, to promote external validity (generalizability), the cluster size was 20 [20]. Kerman University of Medical Sciences’ Ethics Committee (IR.KMU.REC.1400.088.) authorized the study’s procedure. There are no known conflicts of interest and all authors certify responsibility.

### Measures

#### Demographic variables

To calculate the exposure rate, participants were asked to answer yes or no to the question “Were you in Bam when the earthquake happened on December 26?” at the start of the questionnaire. “If not, how long have you lived in Bam?” was the next question. Demographic questions were asked at the end of the data collection. They were also asked whether any of their first-degree relatives died in the 2003 Bam earthquake, to which they replied yes or no.

#### PTGI-SF

The Persian version of the short form of the Posttraumatic Growth Inventory (PTGI-SF) was used to measure PTG [21]. The questionnaire has ten items divided into five subscales (relationships with others, new possibilities, personal strength, spiritual change, and appreciation of life), with two questions in each subscale [22]. The scoring system is a 6-point Likert scale. The global score ranges from zero to 50, and a higher score indicates higher growth.

#### The ASSIST version 3

The ASSIST version 3 had been designed by a group of WHO experts in 2003 [23]. Hooshyari et al. approved the validity and reliability of the Persian version [24]. The researchers used the tool to identify recent consumption of six types of drugs (i.e., tobacco, alcohol, opioids, marijuana, methamphetamine, and sedatives).

### Statistical analyses

Data analysis was done based on chi-square test to compare qualitative characteristics between different groups. Logistic regression analysis was applied to evaluate the true association of variables of interest with the use of various drugs while controlling for potentially confounding factors such as age, gender, education, and exposure to earthquakes. A *p*-value of less than 0.25 in the bivariate analyses was considered as the cut-off for entering the variable in the multivariate logistic regression model Hosmer–Lemeshow (HL) test was used to assess the goodness-of-fit for logistic regression models. *P*-value < 0.05 was considered statistically significant.

## Results

### Descriptive statistics

The study sample included a population of 818 persons (59.6% female, 40.4% male; average age 46.6 ± 11.5 years. Table 1 shows the demographic characteristics of the study participants.

**Table 1:** Demographic characteristics of the sample (*n* = 818)

Variable	*N*	%
**Education**		
Less than college	643	79.3
College	168	20.7
**Marital Status**		
Single	125	15.3
Married	693	84.7
**Job**		
Employed	371	45.4
Retired	109	13.3
Unemployed	338	41.3
**Exposed to earthquake**		
No	244	29.8
Yes	574	70.2
**Death of relatives**		
No	252	31.1
Yes	558	68.9
**PTG**		
None to low PTG	754	92.2
Medium to very high PTG	64	7.8
**Tobacco**		
No	645	78.9
Yes	173	21.1
**Sedatives**		
No	628	76.8
Yes	190	23.2
**Other drugs**		
No	668	81.7
Yes	150	18.3

The prevalence of the three categories of drug consumption according to the demographic characteristics is shown in Table 2. Sedative consumption was comparable in both genders. All of the three categories of drugs were used more frequently by singles than married individuals (*P* < 0.05).

**Table 2:** Prevalence of drug use based on baseline characteristics

Variables		Tobacco	Sedatives	Other drugs
**Gender**	Male	90 (27.4%)	70 (21.3%)	78 (23.8%)
	Female	81 (16.7%)	117 (24.2%)	71 (14.7%)
*P*-value		< 0.001	0.347	0.001
**Education**	Less than college	143 (22.2%)	164 (25.5%)	128 (19.9%)
	College	28 (16.7%)	22 (13.1%)	20 (11.9%)
*P*-value		0.115	0.001	0.017
**Marital status**	Single	43 (34.4%)	50 (40.0%)	31 (24.8%)
	Married	130 (18.8%)	140 (20.2%)	119 (17.2%)
*P*-value		< 0.001	< 0.001	0.043
**Job**	Employed	92 (24.8%)	67 (18.1%)	65 (17.5%)
	Retired	21 (19.3%)	29 (26.6%)	29 (26.6%)
	Unemployed	60 (17.8%)	94 (27.8%)	56 (16.6%)
*P*-value		0.063	0.006	0.054
**Exposed to earthquakes**	Yes	124 (21.6%)	143 (24.9%)	112 (19.5%)
	No	49 (20.1%)	47 (19.3%)	38 (15.6%)
	*P*-value	0.626	0.080	0.183
**Death of close relatives**	Yes	118 (21.1%)	140 (25.1%)	108 (19.4%)
	No	50 (19.8%)	46 (18.3%)	38 (15.1%)
*P*-value		0.671	0.032	0.143
**PTG**	None to low PTG	158 (21.0%)	174 (23.1%)	140 (18.6%)
	Medium to very high PTG	15 (23.4%)	16 (25.0%)	10 (15.6%)
*P*-value		0.641	0.727	0.559

All the *P*-values are reported based on chi-square test

### Logistic regression

For each of the three categories of the drugs, a logistic regression analysis was performed. The results of the three logistic regression models are shown in Tables 3, 4, and 5.

**Table 3:** Logistic regression analysis for determining the effect of baseline characteristics on the consumption of tobacco

Variables	Odds ratio	95% CI	*P* value
**Age**	0.98	(0.97, 1.01)	0.106
**Gender**			
** Male**	1.99	(1.34, 2.95)	0.001
** Female**	Reference	-	-
**Education**			
** Less than college**	1.56	(0.97, 2.51)	0.066
** College**	Reference	-	-
**Marital status**			
** Single**	2.91	(1.81, 4.66)	< 0.001
** Married**	Reference	-	-
**Job**			
** Employed**	Reference	-	-
** Retired**	0.89	(0.48, 1.65)	0.708
** Unemployed**	0.689	(0.44, 1.06)	0.089
**Exposed to earthquakes**			
** Yes**	1.06	(0.70, 1.58)	0.792
** No**	Reference	-	-

**Table 4:** Logistic regression analysis for determining the effect of baseline characteristics on the consumption of sedatives

Variables	Odds ratio	95% CI	*P* value
**Age**	1.02	(1.01, 1.03)	0.048
**Gender**			
** Male**	0.95	(0.64, 1.42)	0.821
** Female**	Reference	-	-
**Education**			
** Less than college**	1.85	(1.10, 3.09)	0.020
** College**	Reference	-	-
**Marital status**			
** Single**	1.91	(1.21,2.99)	0.005
** Married**	Reference	-	-
**Job**			
** Employed**	Reference	-	-
** Retired**	1.18	(0.66, 2.11)	0.566
** Unemployed**	1.46	(0.95, 2.23)	0.082
**Exposed to earthquakes**			
** Yes**	1.33	(0.87, 2.02)	0.182
** No**	Reference	-	-
**Death of close relatives**			
** Yes**	1.056	(0.70, 1.59)	0.798
** No**	Reference	-	-

**Table 5:** Logistic regression analysis for determining the effect of baseline characteristics on the consumption of other drugs

Variables	Odds ratio	95% CI	*P* value
**Age**	0.99	(0.98, 1.02)	0.879
**Gender**			
** Male**	1.92	(1.26, 2.92)	0.002
** Female**	Reference	-	-
**Education**			
** Less than college**	1.86	(1.08, 3.19)	0.024
** College**	Reference	-	-
**Marital status**			
** Single**	1.41	(0.84, 2.38)	0.196
** Married**	Reference	-	-
**Job**			
** Employed**	Reference	-	-
** Retired**	1.78	(0.98, 3.22)	0.056
**Unemployed**	1.07	(0.67, 1.71)	0.767
**Exposed to earthquakes**			
** Yes**	1.16	(0.74, 1.81)	(0.525)
** No**	Reference	-	-
**Death of close relatives**			
** Yes**	1.08	(0.70, 1.68)	0.717
** No**	Reference	-	-

The results in Table 3 showed that the variables of gender and marital status had a significant effect on the consumption of tobacco (*P*-value < 0.05). The chance of consumption of tobacco was 99% higher in males than females and 2.91 times higher in singles than in married after controlling for other variables.

The results in Table 4 showed that the variables of age, education, and marital status had a significant effect on the consumption of sedatives. The chance of consumption of sedatives increased with increasing age and it was about 85% higher in people with less academic education than in people with academic education and 91% higher in singles than in married.

The results in Table 5 showed that the variables of gender and education had a significant effect on the consumption of other drugs. The chance of consumption of other drugs was 92% higher in males than females and 86% higher in people with less academic education than in subjects with academic education after controlling for other variables.

## Discussion

It is essential to identify the factors affecting substance use in order to improve society and reduce the incurred social costs [25]. In the current study, the researchers classified substances into three groups: tobacco, sedatives, and other drugs. Long-term exposure to the Bam earthquake did not significantly correlate with the consumption of any of these substances. According to Maclean et al., exposure to natural disasters at a young age does not increase the risk of narcotic use in adolescence and adulthood [25]. One possibility is that 17 years have elapsed since the earthquake, which has acted as an equal time interval as a risk factor for both groups. Another reason could be the high consumption rate of narcotics in Bam residents before the earthquake. According to Moise and Ruiz, substance abuse disorders remained unchanged before and after Hurricane Katrina [26].

The researchers also found that a rise in age is correlated with an increase in the risk of sedative use, which is consistent with the study by Palamar et al. in 2019 [27]. It has been reported that the severity of insomnia is 30% higher in the elderly [28]. Nevertheless, it has also been suggested that this higher consumption rate is not only due to insomnia treatment; rather, benzodiazepine was prescribed to 20% of people with dementia, which can explain the high rate of sedative use by the elderly [28]. At any rate, due to the simultaneous rise in mortality rate and the use of these medications by the elderly, further studies are warranted.

The researchers found that tobacco and narcotic use is higher in men than women are. Another study after Hurricane Katrina showed that men had more substance abuse disorders than women did. This study also indicated that men had more substance abuse before and after Hurricane Katrina in the studied region [26]. Another possible explanation is that men were unequally exposed to disaster-induced chronic stress, e.g., reduced socioeconomic status and loss of jobs and income, which exacerbated substance use. This justifies the provision of preventive and therapeutic interventions for the target gender groups to maximize the positive outcomes in high-risk regions after disasters [26].

The researchers also found that single people consumed all three groups of substances (tobacco, sedatives, and other substances) more than married people. The literature shows that marriage is a major institution influencing people’s life and welfare. It has also been shown that married people have better physical and mental health. For instance, the level of substance use and depression is lower in married people and they live longer than single people [29]. Two reasons are suggested for this. First, marriage helps promote self-esteem, e.g., it reduces stress in personal or even professional life (having a shoulder to lean on helps personal identity). Second, married people have a higher chance of having a stable and supportive intimate relationship and suffer from loneliness less than single people [29, 30].

The researchers also found that those with lower education levels consume sedatives and other narcotics more than those with a higher level of education. Previous studies have also noted that the level of education affects the risk of narcotic or alcohol abuse. A study in Copenhagen measures the level of education, smoking, and drinking alcohol. It was found that those with the lowest level of education were mostly chain smokers and drank a lot of alcohol [31]. Kumar et al. reported that in New Delhi addiction treatment centers, 21% of the addicted people were illiterate or had only primary education [32]. A higher level of literacy may reduce the tendency to narcotic use due to higher awareness of health literacy. Therefore, it is suggested that the health literacy of society be promoted as an addiction prevention factor.

According to Becker et al., unemployment is directly correlated with the use of sedatives [33]. We also found that the use of sedatives is higher among unemployed people. This could be because unemployed people have a lower income than employed ones. Conway et al. showed that a low-income level is correlated with higher sedative use [34]. Still, more studies on this subject are warranted.

The researchers also showed that sedatives use is higher in those who had lost a loved one in the Bam earthquake. This finding is consistent with previous studies, showing a rise in sedatives and anti-depressant medications post-disaster [35–37]. This could be due to the social context of the earthquake; when many people lose their families, it disrupts the integrity of the social network and causes loss of spirits, irregularity, and disintegration, which negatively affects mental health and, in turn, increases the use of sedatives [35, 38].

## Data Availability

The datasets generated during and/or analyzed during the current study are available from the corresponding author on reasonable request.

## References

[1] Rasmussen T (2004). Macroeconomic implications of natural disasters in the Caribbean.

[2] Rabiei A, Nakhaee N, Pourhosseini SS (2014). Shortcomings in dealing with psychological effects of natural disasters in Iran. Iran J Public Health..

[3] Terri Hurst, Publication, U.N., UNODC. World Drug Report. 2021. 10.1002/9781118929803.ewac0543.

[4] Schonfeld DJ, B. Pediatrics (2011). Ten years after 9/11: what have we (not yet) learned?. J Dev Behav Pediatr..

[5] Pfefferbaum B (2015). Children’s disaster reactions: the influence of exposure and personal characteristics. Curr Psychiatry Rep.

[6] Kopak AM, Van Brown B (2020). Substance use in the life cycle of a disaster: a research agenda and methodological considerations. Am Behav Sci..

[7] Schiff M, Fang L (2016). Adolescents’ exposure to disasters and substance use. Curr Psychiatry Rep..

[8] Bianchini V (2015). PTSD growth and substance abuse among a college student community: coping strategies after 2009 L’aquila earthquake. Clin Pract Epidemiol Ment Health..

[9] Sumner JA (2014). Elucidating dimensions of posttraumatic stress symptoms and their functional correlates in disaster-exposed adolescents. J Psychiatr Res.

[10] Temple JR, Freeman DH (2011). Dating violence and substance use among ethnically diverse adolescents. J Interpers Violence..

[11] Horowitz MJ. Disasters and psychological responses to stress. 1985, SLACK Incorporated Thorofare, NJ. 10.3928/0048-5713-19850301-07.

[12] Arian M (2010). Earthquake-Fault Hazard Investigations In The Kerman Quadrangle.

[13] Talebian M (2006). The Dahuiyeh (Zarand) earthquake of 2005 February 22 in central Iran: reactivation of an intramountain reverse fault. Geophys J Int.

[14] Nemati M (2019). Seismic investigation of 2010 Bardsir and 2011 Sirch and Kahnooj moderate earthquakes in Kerman province. Res Earth Sci..

[15] Berberian M (1984). Field and teleseismic observations of the 1981 Golbaf-Sirch earthquakes in SE Iran. Geophys J Int..

[16] Kenny CJD (2012). Disaster risk reduction in developing countries: costs, benefits and institutions. Disasters..

[17] Amiri H (2021). Posttraumatic growth after earthquake: a systematic review and meta-analysis. Int J Soc Psychiatr..

[18] Kristman V, Manno M, Côté P (2004). Loss to follow-up in cohort studies: how much is too much?. Eur J Epidemiol..

[19] Faul F (2009). Statistical power analyses using G* Power 3.1: Tests for correlation and regression analyses. Behav Res Methods..

[20] Division, U.N.S. Designing household survey samples: Practical guidelines. Vol. 98. United Nations Publications. 2008.

[21] Amiri H, et al. Translation and adaptation of the posttraumatic growth inventory-short form into Persian. 2020;13(1). 10.2174/1874350102013010326.

[22] Cann A (2010). A short form of the Posttraumatic Growth Inventory. Anxiety Stress Coping.

[23] WHO ASSIST Working Group (2002). The alcohol, smoking and substance involvement screening test (ASSIST): development, reliability and feasibility. Addiction..

[24] Hooshyari Z, Sadralssadat J, Sadralssadat L (2013). Estimation of Validation and Reliability of Screening Test of Tobacco Alcohol and Addictive Drugs in Iran.

[25] Maclean JC (2016). Are natural disasters in early childhood associated with mental health and substance use disorders as an adult?. Soc Sci Med..

[26] Moise IK, Ruiz MO (2016). Hospitalizations for substance abuse disorders before and after Hurricane Katrina: spatial clustering and area-level predictors, New Orleans, 2004 and 2008.

[27] Palamar JJ (2019). Shifting characteristics of nonmedical prescription tranquilizer users in the United States 2005–2014. Drug Alcohol Depend.

[28] Hollingworth SA, Siskind DJ (2010). Anxiolytic, hypnotic and sedative medication use in Australia. Pharmacoepidemiol Drug Safety..

[29] Stutzer A, Frey BS (2006). Does marriage make people happy, or do happy people get married?. J Socio-Economics..

[30] Lu L (1999). Personal or environmental causes of happiness: a longitudinal analysis. J Soc Psychol..

[31] Mahajan P, et al. Prevalence of hepatitis-C viral infection among opioid dependent injectable drug users: A study conducted at Swami Vivekananda drug de-addiction and treatment centre, amritsar. 2016;1(2).

[32] Sharma B (2017). Drug abuse: Uncovering the burden in rural Punjab. J Family Med Prim Care.

[33] Becker WC (2007). Non-medical use, abuse and dependence on sedatives and tranquilizers among US adults: psychiatric and socio-demographic correlates. Drug Alcohol Depend..

[34] Conway KP (2006). Lifetime comorbidity of DSM-IV mood and anxiety disorders and specific drug use disorders: results from the National Epidemiologic Survey on Alcohol and Related Conditions. J Clin Psychiatry.

[35] Han K-M (2017). Increase in the prescription rate of antidepressants after the Sewol Ferry disaster in Ansan. South Korea.

[36] Rossi A (2011). A quantitative analysis of antidepressant and antipsychotic prescriptions following an earthquake in Italy. Trauma Stress.

[37] Trifiro G (2013). Effects of L’Aquila earthquake on the prescribing pattern of antidepressant and antipsychotic drugs. Int J Clin Pharm.

[38] Morgan C, Bhugra D (2010). Principles of Social Psychiatry.

